# Excision of Thoracic Chondrosarcoma: Case Report and Review of Literature

**DOI:** 10.7759/cureus.708

**Published:** 2016-07-25

**Authors:** Hai V Le, Rishi Wadhwa, Pierre Theodore, Praveen Mummaneni

**Affiliations:** 1 Orthopedic Surgery, Massachusetts General Hospital; 2 Orthopaedics, Brigham and Women's Hospital; 3 Department of Neurological Surgery, UCSF Medical Center; 4 Department of Thoracic Surgery, UCSF Medical Center

**Keywords:** chondrosarcoma, spine, chest wall, costotransverse, costovertebral, tumor, neuroforamen, thoracic spine, en bloc resection

## Abstract

Chondrosarcomas are cartilage-matrix-forming tumors that make up 20-27% of primary malignant bone tumors and are the third most common primary bone malignancy after multiple myelomas and osteosarcomas. Radiographic assessment of this condition includes plain radiography, computed tomography, and magnetic resonance imaging for tumor characterization and delineation of intraosseous and extraosseous involvement. Most chondrosarcomas are refractory to chemotherapy and radiation therapy; therefore, wide en bloc surgical excision offers the best chance for cure. Chondrosarcomas frequently affect the pelvis and upper and lower extremities. In rare instances, the chest wall can be involved, with chondrosarcomas occurring in the ribs, sternum, anterior costosternal junction, and posterior costotransverse junction. In this article, we present a patient with thoracic chondrosarcoma centered at the left T7 costotransverse joint with effacement of the left T7-T8 neuroforamen. We also detail our operative technique of wide en bloc chondrosarcoma excision and review current literature on this topic.

## Introduction

Chondrosarcomas account for 20-27% of primary malignant bone tumors and commonly affect the pelvis and upper and lower extremities [1]. They are the third most common primary bone malignancy after multiple myelomas and osteosarcomas [2]. Chondrosarcomas can arise de novo (i.e., primary chondrosarcomas) or from malignant transformation of benign cartilage tumors such as enchondromas or osteochondromas (i.e., secondary chondrosarcomas) [3]. The metastatic potential and the surgical prognosis correlate with histologic grading (WHO Grade I to III) [4]. Radiographic assessment of chondrosarcomas includes plain radiography of the entire bone involved. A computed tomography (CT) scan is routinely obtained to better visualize a mineralized chondroid matrix and magnetic resonance imaging (MRI) is also recommended to delineate intraosseous and extraosseous involvement. Because of their hypovascularity and slow mitotic activity, conventional chondrosarcomas are distinguished for their resistance to chemotherapy and radiation therapy [3]. Therefore, wide en bloc surgical excision offers the best chance for cure, especially for Grade II or III chondrosarcomas [1-4].

Although chondrosarcomas are better known for their involvement of the pelvis and extremities, they are the most common malignant tumor of the chest wall [5]. Thoracic chondrosarcomas usually arise from the ribs (costal), ribs and sternum (costosternal), or sternum alone (sternal). Only rarely do they occur at the posterior costotransverse junction (costovertebral) [5, 6]. Extraosseous extension of thoracic chondrosarcomas to the thoracic vertebrae can lead to radiculopathy or myelopathy from nerve root or spinal cord compression, respectively.

In this article, we present a patient with thoracic chondrosarcoma centered at the left T7 costotransverse joint with effacement of the left T7-T8 neuroforamen. We also detail our operative technique of wide en bloc chondrosarcoma excision and review current literature on this topic. Informed consent was obtained from the patient for this case report.

## Case presentation

### History and physical examination

A 45-year-old right-handed male with no known history of cancer presented to our institution with left-sided flank and abdominal pain that persisted for three months. The patient reported concomitant thoracic back pain with numbness and pruritus in the left T7 dermatome but denied experiencing any other symptom of radiculomyelopathy. A physical examination revealed that he had numbness and hyperesthesia in the left T7 dermatomal distribution. A motor exam revealed that the motor system was intact, and the patient had negative long tract signs.

### Imaging studies

**Figure 1:**
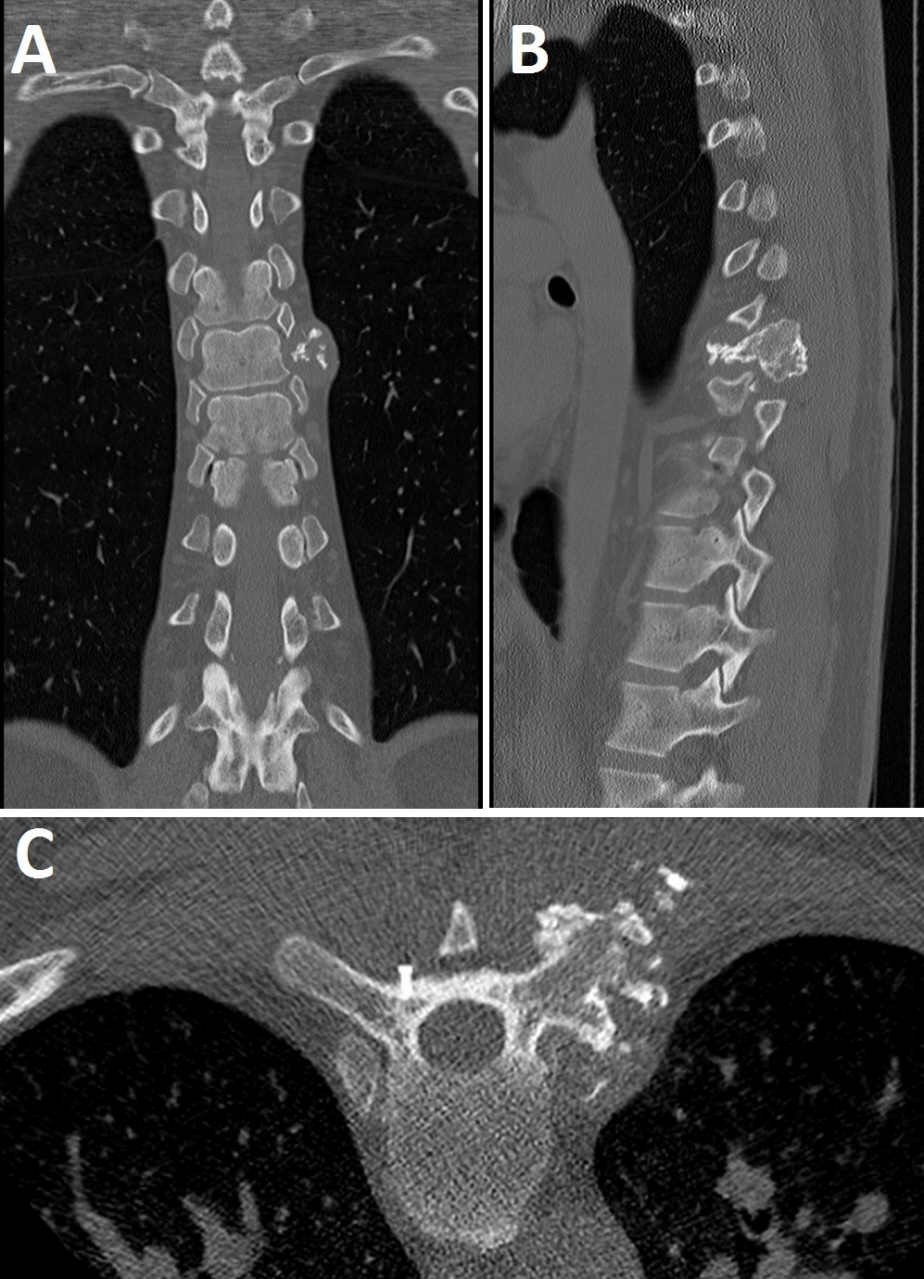
Preoperative CT Imaging Preoperative coronal (A) and sagittal (B) CT images showing a polylobulated mass centered around the left T7 costotransverse joint. A preoperative axial CT image showing fiducial screw placement (C). The fiducial screw is seated within the cortex of the right lamina/transverse junction at the level of the vertebral pedicle, the same level of the involved costovertebral joint contralaterally.

A preoperative unenhanced CT imaging showed a polylobulated mass centered at the left T7 costotransverse joint, measuring approximately 4.8 x 3.3 cm. There was evidence of localized bone destruction as well as an intralesional chondroid matrix. The lesion effaced the left T7-T8 neuroforamen. A preoperative CT scan with coronal (Figure 1A) and sagittal (Figure 1B) reconstructions demonstrated a polylobulated mass with a mineralized chondroid matrix centered at the left T7 costotransverse joint. A fiducial screw was placed percutaneously by interventional radiology for quick and easy identification of the spinal level of involvement during the operation (see Figure 1C) [7].

**Figure 2:**
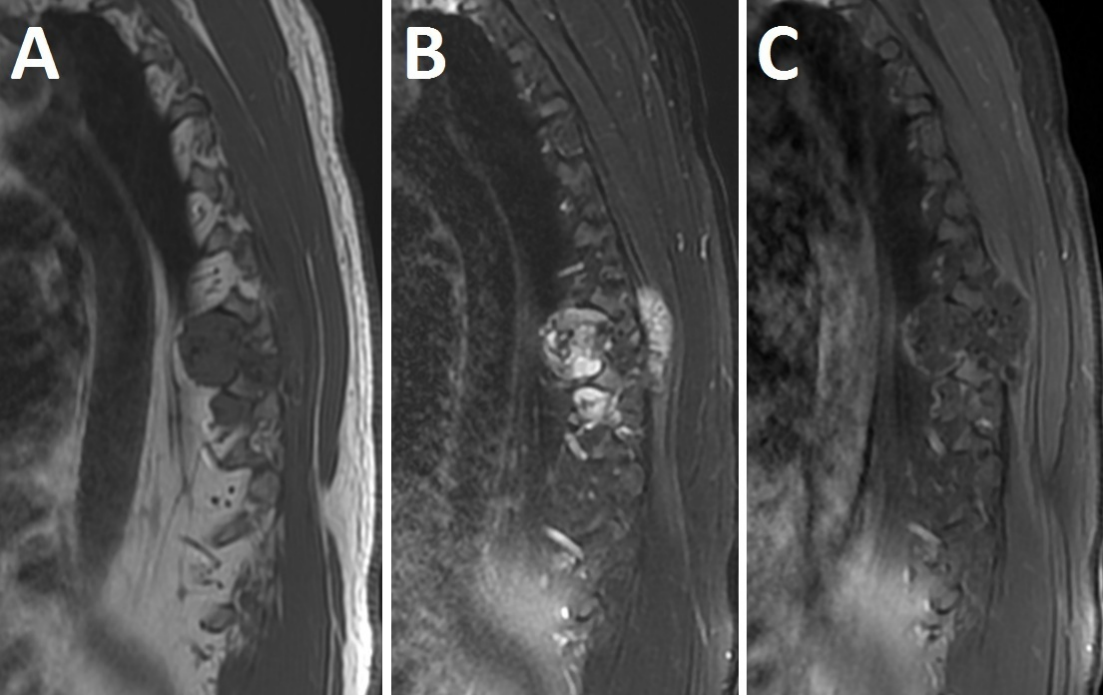
Preoperative MRI Preoperative T1-weighted (A), T2-weighted (B) and T1-weighted gadolinium (C) MRI showing a polylobulated, partially calcified, T2 hyperintense and minimally peripherally enhancing mass centered on the left costovertebral joint at the level of T7 with extensions within the left neuroforamina at T7-8 and T8-9.

A preoperative MRI showed a polylobulated, partially calcified, T2 hyperintense, minimally peripherally enhancing mass centered at the left costovertebral joint, at the level of T7, with extension within the left neuroforamina at T7-T8 and T8-T9 (Figure 2). Following imaging, the patient was taken to the operating room for en bloc resection of the tumor.

### Anesthesia and positioning

The patient was placed under general anesthesia for the procedure. Prophylactic antibiotics and steroids were administered prior to the skin incision. The patient was placed in the prone position on a Jackson table. Draping was done in the normal sterile fashion. Baseline neurophysiological monitoring was performed.

### Iliac crest harvesting

Posterior lateral fusion was planned using an iliac crest autograft. A skin incision on the left side of the pelvis was made with a #15 blade. Subsequently, the crest was harvested with a chisel and gouge. After adequate harvesting, the area was pulse-lavaged thoroughly. Adequate hemostasis was achieved using a bipolar and Surgifoam. The iliac wound was closed in a layered fashion and a drain was tunnelled out of the iliac crest.

### En bloc tumor resection

A midline linear skin incision from T5-T9 was made with a #15 blade. The left tumor area was exposed with blunt dissection. We carefully circumscribed the tumor with blunt dissection without penetrating the tumor itself in order to avoid spillage of tumor tissue. The rib cage was exposed laterally at T7 and T8. The ribs were cut approximately 5 cm lateral from the costotransverse junction in order to get to the lateral aspect around the tumor. The pleura that was attached to the anterior aspect of the T7 and T8 ribs was also removed. Next, the laminae of T5-T9 were exposed for laminectomy. The tumor was found to extend up to the transverse process on the left at T7 and T8. The hard tumor could be palpated through the muscular area. The paraspinal muscles on the left around the tumor were sacrificed in order to achieve en bloc resection. After adequate exposure, laminectomies of the T6-T8 levels were performed. The exiting T7 and T8 nerve roots were sacrificed by tying them off using a 3-0 silk tie and then sectioning them medial to the nerve root ganglion. The neurophysiological motor evoked potentials were checked and found to be stable. A partial left corpectomy at T7 and T8 pedicle and lateral aspect of the vertebral body was performed using a sharp quarter-inch chisel in order to achieve en bloc removal of the tumor. The tumor was then lifted up and removed en bloc.

### Posterior spinal fusion

**Figure 3:**
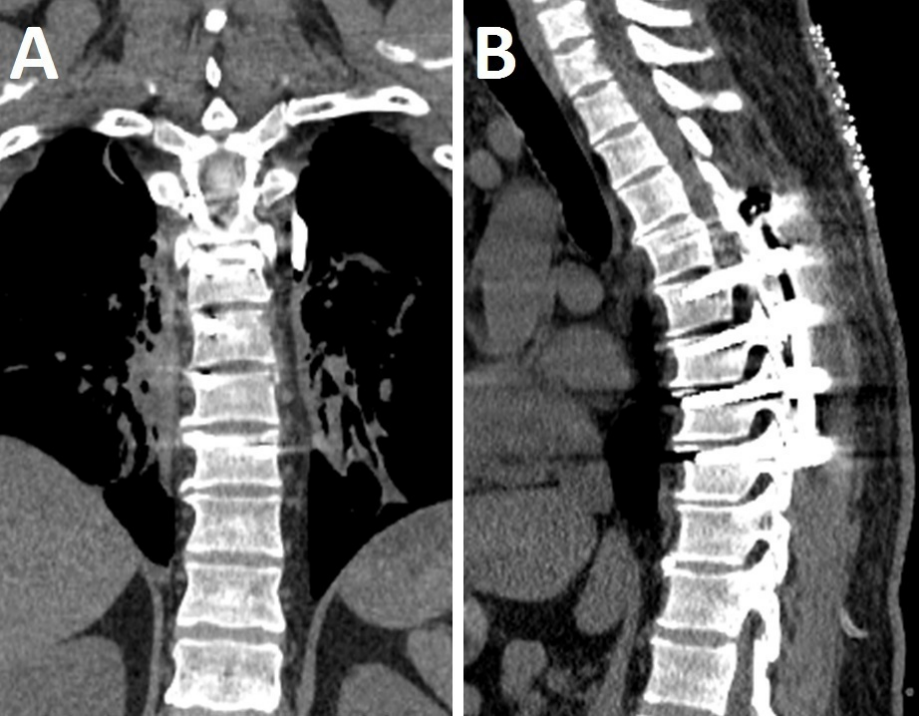
Postoperative CT Imaging Postoperative coronal (A) and sagittal (B) CT images showing postoperative changes consistent with recent T5-T9 posterior spinal fusion. There is no evidence of hardware fracture or loosening.

After en bloc tumor resection, attention was turned to the posterior spinal fusion procedure. The entry points for the pedicle screws from T5-T9 on the right side were decorticated. A gearshift was used to create holes. The pedicle screws were tapped and placed at T5-T9 on the right side. The position of the pedicle screws was checked with the O-arm and adjusted until their placement was satisfactory. On the left side, the pedicle screws were placed at T5, T6, and T9. Since the pedicles at T7 and T8 on the left had been removed, pedicle screws could not be placed there. All of the pedicle screws were 40 mm long and 5.0 mm wide except for the right T6 screw, which was 6.0 mm wide, and the left T9 screw, which was 45 mm long. The rods were cut and contoured. They were then attached into the screw heads. The rods were locked down with locking cap screws. Arthrodesis was achieved by decorticating underneath the rod at T5-T9 on the right side and placing the iliac crest autograft and bone graft extender. After posterior spinal fusion, the surgical site was thoroughly pulse-lavaged. A drain was placed in the chest wall since the lung had been exposed. Hemostasis was achieved with the bipolar. The wound was then closed in a layered fashion. A postoperative CT image obtained revealed the status post T5-T9 posterior spinal fusion instrumentation with no evidence of hardware complications (Figure 3). The estimated blood loss was 1.5 liters.

## Discussion

As seen with our patient, insidious-onset pain is the most common complaint in patients with chondrosarcomas [6]. Hence, chondrosarcomas should be among the leading differential diagnoses in any symptomatic patient who presents with chest wall pain and a palpable mass on clinical exam or a visible mass seen radiographically. As previously stated, chondrosarcomas are the most common malignant tumor of the chest wall [5]. In addition to plain radiography, CT and MRI scans should be obtained to better characterize the tumor. A polylobulated mass with surrounding bone destruction and an intralesional calcified chondroid matrix are distinguishing features of chondrosarcomas on CT imaging. MRI reveals hyperintensity on T2-weighted images, reflecting the high water content of chondrosarcomas [1, 8].

Chondrosarcoma of the chest wall is uncommon compared to the pelvis and extremities. Chest wall chondrosarcomas can occur in the ribs, sternum or both. On rare occasions, they may arise from the posterior costotransverse junction, as illustrated in this case report [5, 6]. Fong, et al. (2004) report on their surgical experience treating 24 patients with chondrosarcoma of the chest wall. The average age of their patient population was 54 years (range 11-76 years). These patients had chondrosarcomas in the ribs (n=16), ribs and sternum (n=2), ribs and spine (n=3), or sternum only (n=3). Histologically, these chondrosarcomas were either Grade 1 (n=17) or Grade 2 (n=7) diseases. From their study, the overall survival estimate at five years was 92%, with improved survival and lower recurrence when adequate surgical margins were achieved [9]. Wiasberg, et al. (2011) report on their experience treating 11 patients (average age 51.5 years), (range 24-74 years) with chondrosarcomas involving the ribs (n=8) or sternum (n=3). One patient had Grade I, eight patients had Grade II and three patients had Grade III chondrosarcomas. The reported recurrence rate and mortality at follow-up were 57% and 50%, respectively [10].

One of the largest reported studies of chest wall chondrosarcomas comes from McAfee, et al. (1985). They evaluated 96 patients with primary chondrosarcoma of the chest wall with a median age of 53.5 years. The location of the chondrosarcomas was as follows: ribs (n=78) and sternum (n=18). Of those, 72 patients underwent treatment at the Mayo Clinic; 28 had wide resection, 25 had local excision, and 19 had palliative excision. Local recurrence occurred in 37 patients (37/72, 51.4%) and of those, 14 patients developed metastatic disease (14/37, 37.8%). In 10 years, recurrence had developed in 50% of patients who had local excision, compared to 17% of patients who had wide resection. Ten-year survival for patients treated with wide resection was 96%, with local excision 65%, and with palliative excision 14%. The authors concluded that survival was influenced by tumor grade, tumor diameter, tumor location, and date of operation [11].

Widhe, et al. (2009) of the Scandinavian Sarcoma Group studied 106 consecutive patients with chest wall chondrosarcomas with a median of nine years follow-up. The patients who had wide surgical resections had a 10-year survival of 92% compared with 47% for those who had intralesional resections. Ten-year survival was also higher in patients treated at sarcoma centers compared to those treated elsewhere (75% versus 59%). As expected, the local recurrence rate was significantly lower when wide resections were achieved (4%) versus intralesional resections (73%). The authors observed that prognostic factors for local recurrence included the surgical margin and histological grade, while prognostic factors for metastases included the histologic grade, tumor size, and local recurrence [12].

Costovertebral chondrosarcomas present an additional challenge to surgical resection. Clinically, extension to the thoracic spine can cause radiculopathy or myelopathy from nerve root or spinal cord compression. In most cases, wide en block surgical resection of chondrosarcomas at the costovertebral joint would require partial or total spondylectomy. Few cases of costovertebral chondrosarcomas have been reported in the literature [13-15]. For chondrosarcomas occupying the costovertebral junction, collaboration between thoracic surgery and spine surgery for wide en block resection followed by reconstruction and stabilization of the thoracic spine offers the best outcomes for patients. We routinely utilized neurophysiological monitoring in these en bloc resections to monitor the functional integrity of the spinal cord and nerve roots intraoperatively. A fiducial screw can be placed percutaneously by interventional radiology for quick and easy identification of the spinal level of involvement during the operation [7]. Alternatively, intraoperative fluoroscopy can serve the same function. When an autograft is anticipated to augment the fusion, we prefer harvesting the autograft first to prevent cross-contaminating normal tissue with tumor tissue. Finally, as previously emphasized, wide surgical margins offer the best chance of cure for patients; therefore, during en block resections, important structures such as nerve roots may have to be sacrificed to achieve this surgical objective.

## Conclusions

Chondrosarcomas are the third most common primary bone malignancy after multiple myelomas and osteosarcomas and is the leading malignant tumor of the chest wall. Thoracic chondrosarcomas usually arise from the ribs (costal), ribs and sternum (costosternal), or sternum alone (sternal); only rarely do they occur at the posterior costotransverse junction (costovertebral). Costovertebral chondrosarcomas can invade nearby nerve roots and the spinal cord, causing radiculomyelopathy symptoms. They present a unique challenge surgically, and collaboration between thoracic surgery and spine surgery is necessary to achieve wide surgical margins followed by reconstruction and stabilization of the thoracic spine.
